# Nitrogen Deposition Reduces the Diversity and Abundance of *cbbL* Gene-Containing CO_2_-Fixing Microorganisms in the Soil of the *Stipa baicalensis* Steppe

**DOI:** 10.3389/fmicb.2021.570908

**Published:** 2021-03-02

**Authors:** Jie Qin, Ming Li, Haifang Zhang, Hongmei Liu, Jianning Zhao, Dianlin Yang

**Affiliations:** Agro-Environmental Protection Institute, Ministry of Agriculture and Rural Affairs, Tianjin, China

**Keywords:** nitrogen deposition, grassland, diversity, *cbbL* gene, CO_2_-fixing microbes

## Abstract

CO_2_ fixation by autotrophic microbes has a significant effect on the carbon cycle in temperate grasslands. Nitrogen (N) deposition in soil has been steadily increasing for decades, which has consequences for soil microorganisms. However, the impact of this deposition on the diversity and abundance of CO_2_-fixing soil microorganisms remains unclear in temperate grasslands. In the present study, the *cbbL* gene, a key gene in the Calvin–Benson–Bassham cycle that encodes the large subunit of ribulose-1,5-bisphosphate carboxylase/oxygenase, was used to study CO_2_-fixing microbes under different rates of N addition (0, 15, 30, 50, 100, and 150 kg N ha^–1^ yr^–1^) in a 9-year field experiment in a temperate grassland. The results showed that N addition led to significant reductions in *cbbL* gene abundance and genetic diversity and altered *cbbL* gene community composition. High N addition enhanced the relative abundances of Acidiferrobacterales and Rhizobiales but reduced those of Burkholderiales and Rhodobacterales. Structural equation modeling further revealed that N addition primarily reduced *cbbL* genetic diversity by increasing the soil NO_3_-N content and decreasing the soil pH. N addition indirectly reduced *cbbL* gene abundance, possibly by increasing the soil N/phosphorus (P) ratio and decreasing the soil pH. These findings suggest that N addition increases the soil available N and causes soil acidification, which may inhibit growth of CO_2_-fixing microbes to some extent.

## Introduction

During the past few decades, the amount of nitrogen (N) deposited from the global atmosphere due to human activities has significantly increased, and developing countries experiencing the most rapid increases (Gruber and Galloway, 2008). Researchers predict that global N deposition will reach 195 Tg N yr^–1^ by 2050 (Galloway et al., 2004). At present, concerns regarding global atmospheric N deposition are primarily focused on Western Europe, North America, and East Asia (mainly China) (Mo et al., 2008). From 1961 to 2008, the proportion of N deposition increased by 59% in China (Lu and Tian, 2014) and reached 10–18 kg N ha^–1^ in the northern grasslands of the country (Zhang et al., 2008; Liu et al., 2011). The rate of atmospheric N deposition is increasing annually and is expected to accelerate in the future (Neff et al., 2002). N deposition changes the physicochemical properties of soil, including with respect to soil acidification and the N–P balance (Hong et al., 2019), altering soil microbial diversity (Wang C. et al., 2018), and affecting the global ecosystem (Penuelas et al., 2013).

The terrestrial biosphere currently absorbs approximately 30% of anthropogenic CO_2_ emissions (Arora and Melton, 2018). In the past, it was generally believed that carbon fixation was primarily dependent on plant photosynthesis and that soil microorganisms contributed to the carbon cycle by participating in the degradation of organic matter rather than by CO_2_ fixation. The role of microorganisms in carbon sequestration may also be underestimated (Hart et al., 2013). Microbial phototrophic CO_2_ fixation accounts for a substantial proportion of global primary productivity (Guzman et al., 2019). However, the contributions of microbial biomass to soil organic matter appear to be much higher than the 1–5% reported by other researchers (Simpson et al., 2007). Lipids, carbohydrates, and proteins have been observed to be produced directly from the CO_2_ taken up by microorganisms (Hart et al., 2013). CO_2_-fixing microorganisms are a group of microorganisms that, like plants, transform atmospheric CO_2_ into organic matter. Soil autotrophic bacteria are important for sequestrating atmospheric CO_2_ (Lynn et al., 2017) and affect the renewal and circulation of organic matter. The CO_2_-fixing capacity of soil microbes has received widespread attention. However, the factors affecting CO_2_-fixing microbes are not well known.

The autotrophic microbial community was previously characterized by targeting the large subunit (encoded by the *cbbL* gene) of form I ribulose-1,5-bisphosphate carboxylase/oxygenase (Rubisco) (Zhao et al., 2018). The *cbbL* gene is an established biomarker that is useful for studying autotrophic bacteria in various soil ecosystems owing to its widespread geographic distribution, functional significance, the increasing number of published sequences of this gene from chemoautotrophs, and its utility in assessing autotrophic microbial diversity in various habitats (Ge et al., 2016; Yue et al., 2019).

The *cbbL*-containing bacteria has gene have been linked to soil available N (Zhou et al., 2019), soil pH (Li M. Q. et al., 2020), and soil organic carbon (SOC) (Xiao et al., 2014; Li et al., 2018). First, increased soil available N may affect *cbbL*-containing bacterial abundance by altering the balance of C/N or N/P ratios (Dong et al., 2019). Second, variations in soil pH are likely to regulate the responses of *cbbL* containing bacteria to environmental changes (Zhao et al., 2018; Li P. P. et al., 2020). Finally, alterations in SOC levels can lead to changes in the abundances of *cbbL*-containing bacteria, particularly upon significant changes in SOC levels (Huang et al., 2018). The relative contribution of these drivers may vary between ecosystems. N deposition is expected to have positive or negative effects on *cbbL* gene-containing CO_2_-fixing microorganisms, which may depend on soil available nutrients and the intensity of N addition, potentially leading to changes in the composition of the *cbbL* gene community. In addition, N deposition can enhance the soil N content and change the soil pH, which in turn may influence the *cbbL* gene-containing CO_2_-fixing microorganisms (Zhou et al., 2019).

Arid and semiarid ecosystems account for approximately 41% of the global land area (Ferrenberg et al., 2015). The vegetative growth of these ecosystems is restricted by many environmental factors, including soil moisture, highlighting the importance of soil CO_2_-fixing microbes (Guo et al., 2015). The *Stipa baicalensis* steppe represents one of the most widely distributed temperate grassland communities in Eurasia and is primarily located on the eastern Mongolian Plateau and in most of the Songliao Plain of China. Although CO_2_-fixing microorganisms make important contributions to ecosystem functions and processes (Zhao et al., 2018), we are only beginning to understand how their communities are shaped by N deposition. In the present study, we conducted field-simulated N deposition experiments to assess the response of soil CO_2_-fixing microbes to different levels of N addition in temperate grassland to address the following two questions: (1) how do the abundance and diversity of *cbbL*-containing bacteria respond to N addition, and (2) what soil environmental factors mediate the response of *cbbL*-containing bacteria to N addition? We hypothesized that N addition affects *cbbL*-containing bacteria by altering soil physicochemical factors (Xiao et al., 2014; Zhou et al., 2019). The results of the present study provide a theoretical basis for the investigation of soil microbial carbon sequestration potential and soil carbon cycles in temperate grasslands. Research on this topic is important for predicting the possible changes in soil carbon sequestration in grassland ecosystems under changes in N deposition.

## Materials and Methods

### Site Description

The field survey was conducted on the *Stipa baicalensis* steppe, which is located in the Hulun Buir grassland (48°30′N, 119°42′E; 765 m) of the Inner Mongolia Autonomous Region, China (Qin et al., 2020). The experimental area has a typical temperate continental monsoon climate with warm summers, cold winters, an annual precipitation of 396 mm and an annual mean temperature of –0.7°C. Most precipitation (66%) in this region occurs in the summer months, and the soil type is primarily a Haplic Calcisol (according to the Food and Agriculture Organization classification). The native vegetation in the study area consists of grasses dominated by species such as *Stipa baicalensis* and *Leymus chinensis*. Common species include *Cleistogenes squarrosa*, *Carex pediformis*, *Filifolium sibiricum*, *Achnatherum sibiricum*, *Thalictrum petaloideum*, *Serratula centauroides*, *Melissitus ruthenica*, and *Carex duriuscula*.

### Experimental Design and Field Measurements

The experimental simulation of N deposition began in 2010, and a randomized block design with four replicates was adopted. Six experimental treatments were performed to simulate current and future N deposition levels such that 24 plots (each 8 m by 8 m) were established. The natural level of N deposition in the Inner Mongolian grassland is approximately 18.1 kg Nha^–1^ yr^–1^ (Zhang et al., 2008). N was added to the six experimental treatments (Stevens et al., 2004; Liu et al., 2011; Lu and Tian, 2014) at 0, 15, 30, 50, 100, and 150 kg N ha^–1^ yr^–1^ (designated as N0, N15, N30, N50, N100, and N150, respectively). N was added twice a year (mid-June and mid-July) by spraying plots with an aqueous solution of NH_4_NO_3_.

### Sampling and Chemical Analyses

Soil sampling was conducted on 10 August 2018 in each of the 24 plots. Before sampling, the litter layer (litter, roots, and stones) was carefully removed. Soil samples were collected from 10 random points across each plot using a soil corer (0–15 cm deep with a 2 cm inner diameter) and mixed to obtain one representative composite sample. The soil samples were placed in self-sealing bags for storage at 4°C. The samples were immediately transported to the laboratory and divided into three subsamples. Subsamples for ammonium N (NH_4_-N) and nitrate N (NO_3_-N) concentration analyses were stored at 4°C for no longer than 1 week, and the subsamples used for soil pH, SOC, and total P (TP) analyses were air dried. The soil physicochemical factors differed under different levels of N addition (Supplementary Table 1). The subsamples used for gene abundance and high-throughput sequencing analyses were stored at –80°C.

### Soil Physicochemical Factors

Soil pH was measured using a soil to water ratio of 1:2.5 using a Delta 320 pH meter (Mettler Toledo Instruments, Shanghai, China). The SOC content was determined with a macro elemental analyzer (Vario MAX C/N; Elementar Analysensysteme, Hanau, Germany). The total N content was determined by Kjeldahl digestion, and the total P concentration was measured using the ammonium molybdate method following H_2_SO_4_-H_2_O_2_-HF digestion. The soil available N (the sum of NH_4_-N and NO_3_-N) was measured with an FIA Star 5000 flow-injection autoanalyzer (Foss Tecator, Höganäs, Sweden).

### Soil *cbbL* Gene Quantification

Genomic DNA was extracted from each soil sample using a PowerSoil DNA Isolation kit (MoBio Laboratories, Carlsbad, CA, United States) according to the manufacturer’s protocol. The integrity and yield of the genomic DNA were assessed by 0.8% agarose gel electrophoresis.

The copy number of the target gene in the DNA samples was determined using the absolute quantitative method. The abundance of the *cbbL* gene was determined by real-time PCR (Applied Biosystems 7900, United States) with the primers K2f (5′-ACCAYCAAGCCSAAGCTSGG-3′) and V2r (5′-GCCTTCSAGCTTG CCSACCRC-3′). Each DNA sample was diluted 10 times, and 2 μl of the diluted DNA (approximately 150 ng of DNA) was then taken as the reaction volume. The 18 μl reaction mixture contained 10 μl of 2 × Taq MasterMix (Takara Bio Inc., Shiga, Japan), 0.5 μl each of the specific forward and reverse PCR primers (Invitrogen, Shanghai, China), and 7 μl of H_2_O. The cycling parameters involved predenaturation at 95°C for 5 min, followed by 30 cycles of 94°C for 30 s, 55°C for 30 s, and 72°C for 30 s, which was followed by a final incubation at 72°C for 10 min. The assays were performed using three technical replicates per sample. A 10-fold dilution series (10^1^10^5^) of plasmid DNA harboring the *cbbL* gene was used to generate a PCR standard curve. At the end of the PCR amplification, the melting curve was analyzed, and a single melting curve peak was observed for each sample. The *cbbL* copies were calculated according to the parameter threshold cycle (Ct) obtained using the 7500 software (version 1.0.6).

### Illumina MiSeq Sequencing of the *cbbL* Gene

Using TruSeq v1/v2 kits (Illumina, San Diego, CA, United States), adaptors A and B, the former of which harbored an 8-nucleotide barcode sequence, were added to the forward and reverse primer sequences, respectively. DNA was detected by 1% agarose gel electrophoresis after genomic DNA extraction. Specific barcoded primers or fusion primers with misplaced bases were synthesized according to the specified sequencing region. The same primer set (K2f [5′-ACCAYCAAGCCSAAGCTSGG-3′] and V2r [5′-GCCTTCSAGCTTG CCSACCRC-3′]) and a thermal profile for real-time PCR were used for *cbbL* gene amplification with Illumina MiSeq sequencing. The PCR products of the same sample were mixed and detected by 2% agarose gel electrophoresis. Then, the PCR products were recovered via gel extraction using an AxyPrep DNA gel recovery kit (Axygen Inc., Union City, CA, United States) and a Tris-HCl elution and were detected by 2% agarose electrophoresis. All the PCR steps were performed with a Mastercycler Gradient (Eppendorf, Hamburg, Germany). The resulting purified amplicons were pooled in equimolar concentrations and paired-end sequenced on an Illumina MiSeq PE300 platform (Illumina, San Diego, CA, United States) by Allwegene Technology Co., Ltd. (Beijing, China). The raw sequence data were submitted to the National Center for Biotechnology Information Sequence Reads Archive under accession No. PRJNA633225.

The *cbbL* gene sequences were checked for close relatives to known *cbbL* sequences in GenBank (the National Center for Biotechnology Information database) using the BLAST program^1^. Trimmomatic (version 0.36) and PEAR (version 0.96) were used to manipulate the FASTQ data. The sliding-window strategy was adopted using a window size set to 50 bp, an average mass value of 20, and a minimum reserved sequence length of 120. FLASH (version 1.20) and PEAR were used to merge the two end sequences according to the overlap relationship of the PE. The minimum overlap was set to 10 bp, and the mismatch rate was 0.1 to obtain the FASTA sequence. The raw data were screened, and sequences were removed from consideration if they were shorter than 200 bp, had a low-quality score (≤20), contained ambiguous bases, or did not exactly match primer sequences and barcode tags. Clean tags were clustered (or denoised) to generate operational taxonomic units (OTUs) using the UPARSE method (version 9.2) (Edgar, 2013) and the UNOISE method (Rognes et al., 2016). The sequences were clustered into OTUs at a similarity level of 97% (Edgar, 2013), resulting in the identification of 1542 OTUs, from which 1528 OTUs were extracted. A rarefaction curve was constructed by the random sampling of sequences and the number of OTUs that they represented (Amato et al., 2013). Sample rarefaction curves can be used to check the rationality of the data (Supplementary Figure 1). If the curve tends to be flat, more data will only generate a small number of new OTUs, indicating that the amount of sequencing data used is reasonable. The Shannon–Wiener curve (Edgar, 2013) is an index that reflects the diversity of microorganisms in a sample and is constructed using the microbial diversity index of each sample at different sequencing depths to reflect the microbial diversity of each sample with different sequencing quantities (Supplementary Figure 2). If the curve tends to be flat, the amount of sequencing data is large enough to reflect most of the microbial information in the sample. The alpha diversity indices of the *cbbL*-harboring bacteria were calculated with MOTHUR software at a 97% similarity based on the OTU clustering results.

### Data Processing and Analysis

ANOVA was performed to test the effects of the N addition gradient on *cbbL* gene abundance, *cbbL* OTUs, Shannon index values, bacterial phospholipid-derived fatty acids (PLFAs) and dominant *cbbL*-containing bacteria using IBM SPSS 20.0. Fisher’s least significant difference (LSD) multiple range test was used to determine the significance of differences among the N addition treatments.

Correlation analysis was used to assess how *cbbL* gene abundance and *cbbL* OTUs were related to the Shannon index using IBM SPSS 20.0.

We estimated the strength of the relationships between N addition and NO_3_-N and NH_4_-N contents, the N/P ratio, pH, *cbbL* gene abundance and *cbbL* diversity by structural equation modeling (SEM) using IBM AMOS 21.0. In this model, we hypothesized that N addition may directly alter the abundance and diversity of the *cbbL* gene and indirectly change the NO_3_-N and NH_4_-N contents, N/P ratio and pH value. We used the chi-square test (χ^2^), goodness-of-fit index (GFI), root mean square error of approximation (RMSEA) and Akaike information criterion (AIC) to assess the degree of fit of this model.

The overlap of the number of OTU groups under different N addition treatments is represented by a Venn diagram. Principal coordinate analysis (pCoA) was used to visualize the differences in *cbbL* gene communities among the different N addition treatments using the R software environment (version 3.6.1). To determine if N addition altered *cbbL* gene community composition, a permutational analysis of variance (PERMANOVA) analysis was conducted with the Bray–Curtis similarity index using the R software environment (version 3.6.1). The biomarkers with significant differences in abundance between groups under the different N addition treatments were identified by linear discriminant analysis effect size (LEfSe; score = 3).

## Results

### Effect of Nitrogen Deposition on *cbbL* Gene Abundance and Diversity

**FIGURE 1:**
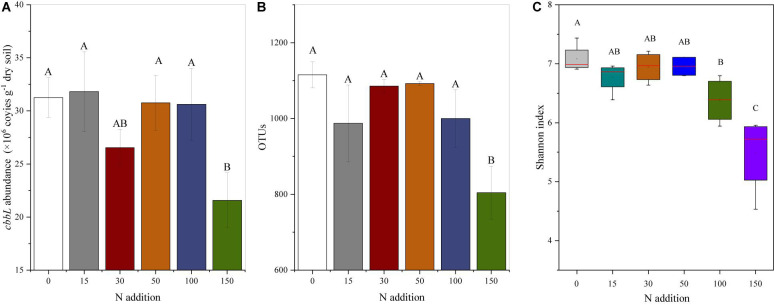
Effects of N addition on **(A)** *cbbL* gene abundance, **(B)** OTU abundance, and **(C)** diversity. The error bars show one standard error of the mean **(A,B)**. The central mark in each box indicates the average value, the central line indicates the median, and the bottom and top edges of the box indicate the 25th and 75th percentiles, respectively. The whiskers extend to the most extreme data points that are not considered outliers, and the outliers are labeled with the ‘+’ symbol **(C)**. Different letters above the bars indicate significant differences based on the LSD multiple range test (*P* < 0.05).

The results showed that compared to the N0 treatment, the N150 treatment reduced the *cbbL* gene abundance, with an observed decrease of 8% (Figure 1A and Supplementary Table 2), whereas no differences were observed among the N0-N100 treatments. The OTUs decreased with increased N addition. Compared to the N0 treatment, the N150 treatment significantly reduced the OTUs by 9% (Figure 1B and Supplementary Table 2), whereas no significant differences were observed among the N0-N100 treatments. N addition significantly affected the Shannon index (*P* < 0.001; Figure 1C and Supplementary Table 2). Compared to that of the N0 treatment, the Shannon index decreased by 10.0 and 22.69% under the N100 and N150 treatments, respectively, whereas no differences were observed among the N0–N50 treatments. No significant differences were observed in the other *cbbL* genetic diversity indices (Chao1, observed species, and PD whole tree) with different N addition levels (*P* > 0.05; Supplementary Table 2). The results showed that compared to the N0 treatment, the N100 treatment enhanced the bacterial PLFAs, with an increase of 75% (Supplementary Figure 3 and Supplementary Table 2).

### Effect of Nitrogen Deposition on the Composition of the *cbbL* Gene Community

**FIGURE 2:**
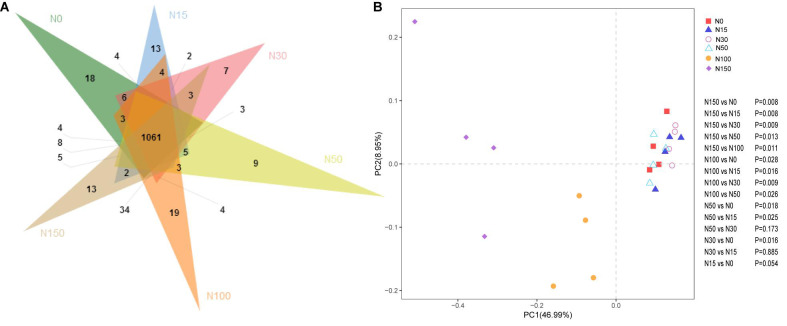
Effects of N addition on *cbbL* gene community composition. **(A)** Venn diagram of different N addition treatments. Different colors represent different N addition rates. The overlapping areas of the different colors represent the intersections (i.e., the common OTUs occur in areas where the color overlap, whereas the non-overlapping areas show unique OTUs). **(B)** Principal coordinate analysis (weighted PCoA) of *cbbL* gene communities based on Bray–Curtis dissimilarity matrices across different N addition treatments. Different shapes represent different N addition treatments. The percent variation explained by the plotted principal coordinates is indicated on the axes. Pairwise PERMANOVA results are displayed to the right of the PCoA. The *cbbL*-containing microbiota from the N100 (orange circles) and N150 (purple diamond) treatments clustered separately from *cbbL*-containing microbiota of the N0–N50 treatments (*P* < 0.05 by PERMANOVA).

Under the different levels of N addition, 1061 common OTUs were observed, representing 69.7% of the total OTUs. The N0, N15, N100, and N150 treatments had 18, 13, 19, and 13 specific OTUs, respectively (Figure 2A). The PCoA results showed that the *cbbL* gene communities clustered strongly based on different N addition levels (Figure 2B). The PERMANOVA results for the *cbbL* gene communities was consistent with those of the PCoA and could explain 46.99% of the variation in the *cbbL*-containing bacterial communities under different N addition levels. Specifically, the first coordinate (PCoA1) separated the N100 and N150 treatments from the other N treatments (N0, N15, N30, and N50), while the second coordinate (PCoA2) explained the remaining 8.95% of the dissimilarity.

**FIGURE 3:**
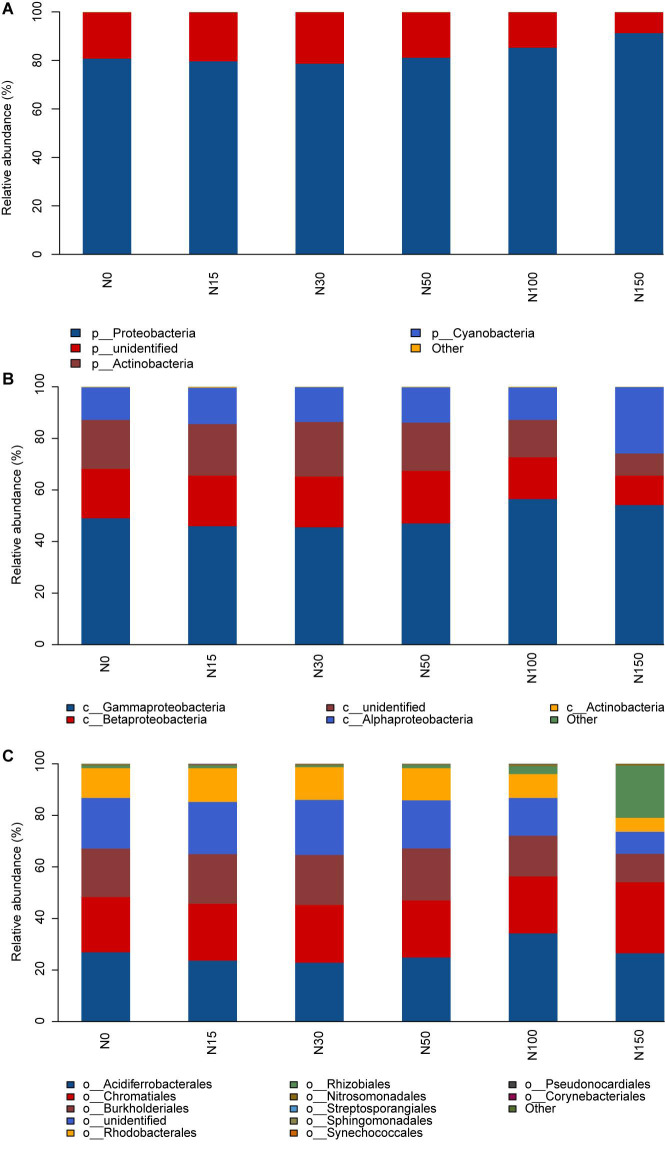
Relative abundances of the dominant phyla **(A)**, classes **(B)**, and orders **(C)** under different levels of N addition.

At the phylum level, members of the phylum Proteobacteria were the dominant *cbbL*-containing bacteria (relative abundance > 80%), the relative abundance of which increased with increasing N addition (*P* < 0.001; Figure 3A and Supplementary Table 2). The results showed that compared to the N0 treatment, the N150 treatment enhanced the relative abundance of Proteobacteria, with an observed increase of 11% (Supplementary Table 3), whereas no differences were observed among the N0–N50 treatments.

At the class level, Gammaproteobacteria (45.4–56.2% relative abundance), Betaproteobacteria (11.25–20.25% relative abundance), and Alphaproteobacteria (12.75–25.75% relative abundance) were the dominant *cbbL*-containing bacteria (Figure 3B). The relative abundances of Gammaproteobacteria and Alphaproteobacteria were enhanced with increasing N addition (*P* < 0.01; Figure 3B and Supplementary Tables 2, 3). Compared to the N15 treatment, the N150 treatment significantly increased the relative abundance of Gammaproteobacteria by 8%. Compared to the N0 treatment, the N150 treatment significantly increased the relative abundance of Alphaproteobacteria by 13%. The relative abundance of Betaproteobacteria was significantly decreased under the N100 and N150 treatments, whereas no differences were observed among the N0–N50 treatments (*P* < 0.001; Supplementary Table 3).

At the order level, Acidiferrobacterales (23.00–34.25% relative abundance), Chromatiales (21.5–27.5% relative abundance), Burkholderiales (11.00–20.00% relative abundance), Rhizobiales (1.0–20.00% relative abundance), and Rhodobacterales (5.50–13.00% relative abundance) were the dominant *cbbL*-containing bacteria (Figure 3C). The N100 treatment significantly increased the relative abundance of Acidiferrobacterales, whereas no differences were observed among the N0–N50 and N150 treatments. Compared to the N0 treatment, the N150 treatment significantly increased the relative abundance of Rhizobiales by 19%, whereas no differences were observed among the N0–N100 treatments. The relative abundance of Burkholderiales and Rhodobacterales was significantly decreased in the N100 and N150 additions, whereas no differences were observed among the N0–N50 treatments (*P* < 0.001; Figure 3C and Supplementary Tables 2, 3).

The structure of the *cbbL* -containing gene community changed under N addition in 10 families (Supplementary Figure 4). Under the N100 treatment, significant differences in *cbbL*-containing microbes occurred in the families Acidiferrobacteraceae (order Acidiferrobacterales) and Sphingomonadaceae (order Sphingomonadales). Under the N150 treatment, a significant difference in *cbbL*-containing microbes occurred in the family Bradyrhizobiaceae (order Rhizobiales).

### Factors Affecting *cbbL*-Containing Microbes

The correlation analysis results showed that the Shannon index was positively correlated with *cbbL* gene abundance (*r*^2^ = 0.464, *P* = 0.022; Supplementary Figure 5). The number of OTUs was positively correlated with *cbbL* gene abundance (*r*^2^ = 0.620, *P* = 0.001; Supplementary Table 4). The abundance of *cbbL*-containing microbes was significantly correlated with soil properties. The *cbbL* gene abundance (*r*^2^ = 0.473, *P* = 0.002; Supplementary Table 5) and the Shannon index were negatively correlated with total N (*r*^2^ = 0.541, *P* = 0.006; Supplementary Table 4), while the Shannon index was negatively correlated with NO_3_-N (*r*^2^ = 0.596, *P* = 0.002; Supplementary Table 5) and NH_4_-N (*r*^2^ = 0.521, *P* = 0.009; Supplementary Table 5). A positive correlation was observed between pH and the number of OTUs (*r*^2^ = 0.443, *P* = 0.03; Supplementary Table 5), *cbbL* gene abundance (*r*^2^ = 0.442, *P* = 0.031; Supplementary Table 5) and the Shannon index (*r*^2^ = 0.634, *P* = 0.001; Supplementary Table 5). In addition, the *cbbL* gene abundance (*r*^2^ = 0.460, *P* = 0.024; Supplementary Table 5) and the Shannon index (*r*^2^ = 0.598, *P* = 0.002, Supplementary Table 4) were negatively correlated with the N/P ratio. There was a positive correlation between C/N and the number of OTUs (*r*^2^ = 0.438, *P* = 0.032; Supplementary Table 5), *cbbL* gene abundance (*r*^2^ = 0.552, *P* = 0.005; Supplementary Table 5) and the Shannon index (*r*^2^ = 0.606, *P* = 0.002; Supplementary Table 5). No correlations were observed between *cbbL*-containing microbes and SOC levels (Supplementary Table 5).

**FIGURE 4:**
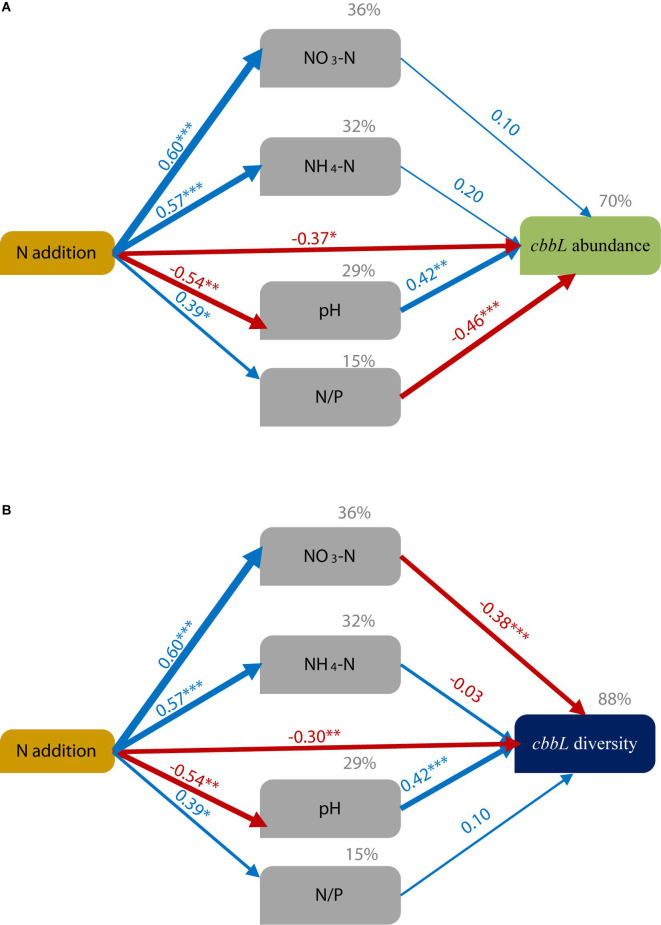
Structural equation modeling (SEM) of the effects of N addition on soil physicochemical factors (pH, NO_3_-N, NH_4_-N, and the N/P ratio) and CO_2_-fixing microbes [*cbbL* gene abundance **(A)** and *cbbL* diversity **(B)**]. The final model resulted in a good fit to the data. ***P ≤ 0.001, **P ≤ 0.01, *P ≤ 0.05. For *cbbL* gene abundance: χ^2^ = 6.575, df = 6, *P* = 0.362; RMSEA = 0.065; GFI = 0.919 and AIC = 36.575. For *cbbL* diversity: χ^2^ = 6.575, df = 6, *P* = 0.362; RMSEA = 0.065; GFI = 0.919; and AIC = 36.575. The number above the arrow indicates the standardized path coefficient, and the width of the arrow indicates the effect size of the relationship. The percent (gray) variance explained (*R*^2^) is shown above each variable. The blue arrows indicate a significant positive relationship (*P* < 0.05), while the red arrows indicate a negative relationship (*P* < 0.05).

The SEM results explained 70% of the variation in *cbbL* gene abundance (Figure 4A and Supplementary Table 6) and showed that N addition indirectly affected the *cbbL* gene abundance by altering the soil N/P ratio and soil pH. However, the contribution of available N was highly limited. The SEM explained 88% of the variation in the diversity of *cbbL*-containing microbial OTUs. In addition, the SEM results (Figure 4B and Supplementary Table 6) showed that N addition indirectly affected the diversity of *cbbL*-containing microbes by altering the soil NO_3_-N content and soil pH.

## Discussion

In the present study, we investigated the responses of CO_2_-fixing microbes to N additions through a 9-year controlled experiment conducted in temperate grassland. Three primary results emerged: (1) N addition reduced the *cbbL* gene abundance and the diversity of *cbbL*-containing microbes; (2) the decrease in *cbbL*-containing microbial diversity was associated with an increase in NO_3_-N content and pH; and (3) N deposition reduced *cbbL* gene abundance, possibly by altering the soil pH and N/P ratio.

Treseder (2008) previously observed that microbial biomass decreased by 15% under N deposition, indicating that atmospheric N deposition would inhibit the growth and reproduction of soil microorganisms in 28 regions worldwide. Our results demonstrated that high N addition reduced both *cbbL* gene abundance and diversity. Research findings on the effects of N addition on microbial diversity have been inconsistent. The results of a previous meta-analysis revealed that N addition, particularly high N addition (above 100 kg N ha^–1^ yr^–1^), decreased soil microbial diversity, although the effects may vary among different ecosystems (Wang C. et al., 2018). In contrast, Zhou et al. (2019) observed that *cbbL* gene diversity was lowest under 0N addition conditions and that there was a positive effect of nutrient addition on soil *cbbL* gene diversity after 26 years of fertilization. Under long-term field fertilization, an increase in soil available nutrients has been shown to promote *cbbL* gene abundance and diversity (Yuan et al., 2012). Our results showed that high N addition (150 kg N ha^–1^ yr^–1^) decreased the *cbbL* gene abundance and diversity in temperate grasslands after 9 years of N addition. Thus, the different response patterns among various plant community compositions and long-term fertilization may depend on the ecosystem environment.

The decreased *cbbL* gene abundance and diversity were observed to be linked to changes in soil properties, particularly the concentration of NO_3_-N, the soil pH and the N/P ratio, associated with N addition. As N addition increased, the NO_3_-N content and the N/P ratio increased significantly, whereas the soil pH decreased significantly (Supplementary Table 1). The soil pH decreased with the increased addition of NO_3_-N, which is consistent with the results of previous studies (Zeng et al., 2016; Wang Q. et al., 2018; Hong et al., 2019). Zhao et al. (2018) also observed that *cbbL* gene abundance was significantly correlated with soil pH. N addition has been shown to result in an imbalance in the soil N/P ratio in terrestrial ecosystems (Vitousek et al., 2010; Li et al., 2016). The soil N/P ratio appeared to have a negative effect on *cbbL* gene abundance, with the N/P ratio being the key influencing factor. The soil available N content was previously shown to be significantly correlated with the gene abundance of CO_2_-fixing microorganisms (Huang et al., 2018; Du et al., 2019). However, the contributions of NH_4_-N and NO_3_-N contents to the *cbbL* gene abundance were limited under N addition conditions in our present study.

Nutrient availability can alter microbial diversity (Zhang et al., 2012). Our results showed that N addition primarily reduced the diversity of *cbbL*-containing microbes by altering the soil NO_3_-N concentration and pH. Zhao et al. (2018) previously reported that high concentrations of available N increase the diversity of CO_2_-fixing microorganisms. These results contrast with our findings, which indicated that high NO_3_-N concentrations suppress the diversity of CO_2_-fixing microorganisms. Soil microorganisms are becoming increasingly vulnerable due to increasing N deposition in grasslands (Wang C. et al., 2018).

N addition consistently altered *cbbL* gene community composition. The order Acidiferrobacterales was previously identified as the dominant group of CO_2_-fixing of microbes in temperate grassland, while Rhizobiales became the dominant order under high N addition. Zhou et al. (2019) observed that Proteobacteria was the dominant known soil *cbbL*-containing bacterial phylum with the highest abundance after 26 years of fertilization. Consistent with their results, we observed that the relative abundance of Proteobacteria was greater than 80% and increased with N addition. Compared to low and intermediate rates of N addition, the CO_2_-fixing microbial community characteristics were different under the N100 and N150 treatments. The relative abundances of Acidiferrobacterales and Rhizobiales were higher in the N100 and N150 treatments than those observed in the other treatments. Consistent with our results, Zhang et al. (2017) observed that the relative abundance of Acidobacteria increased at a high N level. The response of Acidiferrobacterales is primarily caused by N addition and pH changes (Ramirez et al., 2012). Our results showed that members of the order Rhizobiales became the dominant CO_2_-fixing microbes under the N150 treatment (from a 1% relative abundance under N0 addition to a 20.25% relative abundance under N150), which might play a significant role in microbial CO_2_ fixation. Research on grassland and forest ecosystems has also demonstrated that Rhizobium is the dominant bacterium among carbon-fixing microorganisms (Guo et al., 2015; Li et al., 2018; Zhao et al., 2018). The dominant populations of CO_2_-fixing microorganisms in different research areas vary greatly and are ultimately determined by soil properties and genetic characteristics (Tolli and King, 2005).

Soil organic carbon is one of the most significant factors influencing the abundance and diversity of *cbbL*-containing bacteria (Yuan et al., 2012; Xiao et al., 2014; Li et al., 2018), but our results indicated that SOC contributed little to the changes in CO_2_-fixing microbes under N addition. Research shows that the SOC concentration, particularly significant changes in SOC concentration, has a significant relationship with the diversity and abundance of *cbbL*-containing bacterial communities (Osborn et al., 2000; Yuan et al., 2012). In our present study, N addition did not alter the SOC concentration (Supplementary Table 1). Another N deposition study also reported no impact of N addition on SOC (Schleuss et al., 2019). Such patterns suggest that the contribution of SOC to CO_2_-fixing microbes is highly limited under the N addition conditions studied. This inconsistency in the results suggests that other important factors besides SOC may trigger changes in CO_2_-fixing microbes. We also considered other factors that could potentially impact CO_2_-fixing microbes in response to N addition, such as the N/P ratio. Several previous studies have reported that the N/P ratio, particularly under P deficiency, is crucial under N addition conditions and impacts CO_2_-fixing microbes (Dong et al., 2019). Consistent with their results, we observed significant negative correlations between the N/P ratio and the abundance of CO_2_-fixing microbes.

In the present study, the coregulatory mechanism of soil available N and pH is the most likely factor responsible for the observed loss of the abundance and diversity of CO_2_-fixing microbes under high N addition. First, N deposition can influence the soil available N concentration (Liu et al., 2013), where high NO_3_-N levels may inhibit CO_2_-fixing microbial activity. The increased concentration of NH_4_-N did not significantly influence CO_2_-fixing microbial diversity. Since effects of NH_4_-N and NO_3_-N on soil CO_2_-fixing microbial diversity have been observed, with the increasing proportion of NO_3_-N in N deposition, the impact of N deposition on soil CO_2_-fixing microbiota may become more severe (Liu et al., 2013). However, observations on the effects of soil NH_4_-N were from one grassland ecosystem, and whether the same phenomenon will happen in other systems should be further studied. Second, the significant positive correlation between the changes in the abundance and diversity of CO_2_-fixing microbes and the changes in soil pH and SEM results both indicated a link between CO_2_-fixing microbes and soil pH. Soil pH changes in soil co-occur with interactions among soil available nutrition (e.g., available N) (Lammel et al., 2018), masking many indirect effects of pH on soil CO_2_-fixing microbes. High levels of N addition can drive soil acidification both directly and indirectly (Guo et al., 2010). The consequence of soil acidification is the destruction of microecological balance, which leads to the loss of a stable and healthy soil environment (Wan et al., 2020) and is unfavorable for CO_2_-fixing microbial growth.

## Conclusion

To the best of our knowledge, this is the first study to report the negative effects of N addition on the CO_2_-fixing microbes present in temperate grassland. We showed that N addition primarily reduced the abundance and diversity of CO_2_-fixing microbes by altering soil available N and soil pH. These findings suggest that N addition can alter CO_2_-fixing microbes and indicate the importance of the coregulation of soil available N and pH under N addition. The contribution of SOC to alterations in the various CO_2_-fixing microbes under increased N addition was very limited. As different effects of NH_4_-N and NO_3_-N on soil CO_2_-fixing microbial diversity were observed in the present study, we further speculate that with the increasing proportion of NO_3_-N in N deposition, the impact of N deposition on soil CO_2_-fixing microbiota may become more severe.

## Data Availability Statement

The datasets presented in this study can be found in online repositories. The names of the repository/repositories and accession number(s) can be found in the article/Supplementary Material.

## Author Contributions

DY conceived and designed the experiments. ML, HZ, JZ, and HL performed the experiments. JQ analyzed the data. JQ and DY wrote the manuscript. All authors approved the final manuscript.

## Conflict of Interest

The authors declare that the research was conducted in the absence of any commercial or financial relationships that could be construed as a potential conflict of interest.

## Footnotes

1 http://blast.ncbi.nlm.nih.gov/Blast.cgi

## Supplementary Material

Supplementary Figure 1 Sample rarefaction curves. F, Fo, S, and T represent the experimental replicates.

Supplementary Figure 2 Shannon–Wiener curves. F, Fo, S, and T represent the experimental replicates.

Supplementary Figure 3 Effects of N addition on the bacterial Plfas. The different letters above bars indicate significant differences based on the Lsd multiple range test (*P* < 0.05). The central mark in each box indicates the average value, the central line indicates the median, and the bottom and top edges of the box indicate the 25th and 75th percentiles, respectively. The whiskers extend to the most extreme data points that are not considered outliers, and the outliers are labeled with the ‘+’ symbol.

Supplementary Figure 4 Cladogram showing carbon dioxide-fixing microbial community compositions under different N addition treatments (LefSe; score = 3). The six rings of the cladogram represent the domain (innermost) phylum, class, order, family, and genus. The enlarged circles in dark green, blue, and red are differentially abundant taxa identified as taxonomic biomarkers under the different treatments. Lineages with linear discriminant analysis scores with a threshold value of 3.0 were used to identify the degree of differentiation between treatments.

Supplementary Figure 5 Relationship between the *cbbL* Shannon index values and abundance. The solid red line indicates a significant relationship, and the shaded area shows the 95% confidence interval of the fit.

Supplementary Table 1 Soil physicochemical factors under different N addition levels.

Supplementary Table 2 Results (*F* values) of an Anova of the effects of N addition on bacterial Plfas, *cbbL* gene abundance, Otus, the Shannon index, and the relative abundance of dominant *cbbL*-containing microbes.

Supplementary Table 3 Soil *cbbL*-containing microbes under different N addition levels.

Supplementary Table 4 Correlation analysis between the bacterial Plfas and *cbbL* Otus, abundance, and Shannon index values.

Supplementary Table 5 Correlation analyses between soil properties and the *cbbL* Otus, abundance, and Shannon index values.

Supplementary Table 6 Structural equation modeling of the effect of N addition on the *cbbL* diversity (Otus) and abundance through all plausible interaction pathways. The tables show the unstandardized path coefficients (estimates), standard error of regression weights (S.E.), critical values for the regression weights (C.R.), and levels of significance of the regression weights (*P*). ^∗∗∗^ Indicates *P* ≤ 0.001, ^∗∗^ indicates *P* ≤ 0.01, and ^∗^ indicates *P* ≤ 0.05.
