# Supplementary material for: Nitrogen Deposition Reduces the Diversity and Abundance of cbbL Gene-Containing CO2-Fixing Microorganisms in the Soil of the Stipa baicalensis Steppe
Source: Front Microbiol. 2021 Mar 2;12:570908. doi: 10.3389/fmicb.2021.570908 (PMC7961154; doi:10.3389/fmicb.2021.570908)
Supplement: Supplementary Figure 1 — Sample rarefaction curves. F, Fo, S, and T represent the experimental replicates. [file Data_Sheet_1.pdf]

Nitrogen deposition reduces the diversity and abundance of *cbbL* gene-containing CO_2-fixing microorganisms in the soil of the *Stipa baicalensis* steppe

Jie Qin, Ming Li, Haifang Zhang, Hongmei liu, Jianning Zhao* and Dianlin Yang*

Agro-Environmental Protection Institute, Ministry of Agriculture and Rural Affairs, Tianjin, China.

E-mails: qinjie@caas.cn (Jie Qin); 1364805890@qq.com (Ming Li); hfzhang12@126.com (Haifang Zhang); liuhongmei@caas.cn (Hongmei Liu); zhaojianning@caas.cn (Jianning Zhao); yangdianlin@caas.cn (Dianlin Yang).

*Corresponding authors: Agro-Environmental Protection Institute, Ministry of Agriculture and Rural Affairs, 211, Fukang Road, Nankai District, Tianjin 300191, China. E-mail addresses: zhaojianning@caas.cn (Jianning Zhao); yangdianlin@caas.cn (Dianlin Yang), Tel.: +00-86-022-23611820.

Supplementary Material

1.1 Supplementary Figures

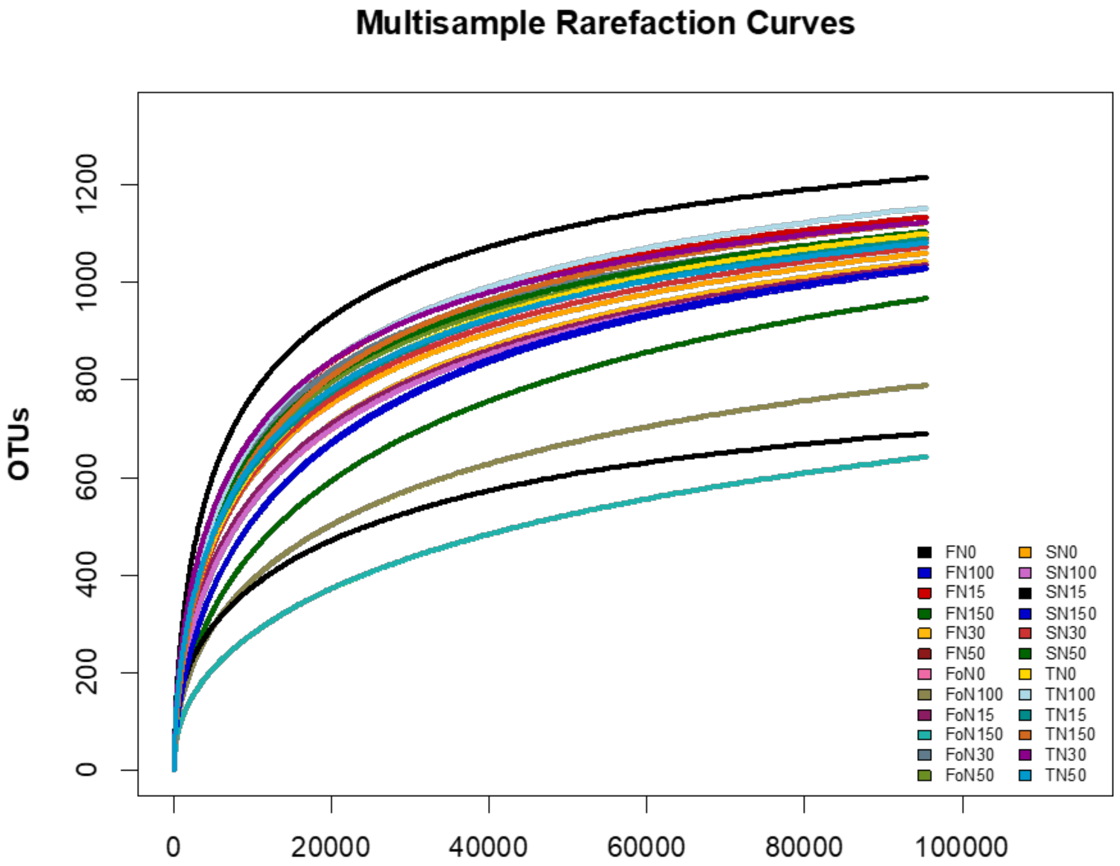

Supplementary Fig. 1 Sample rarefaction curves. F, Fo, S, and T represent the experimental replicates.

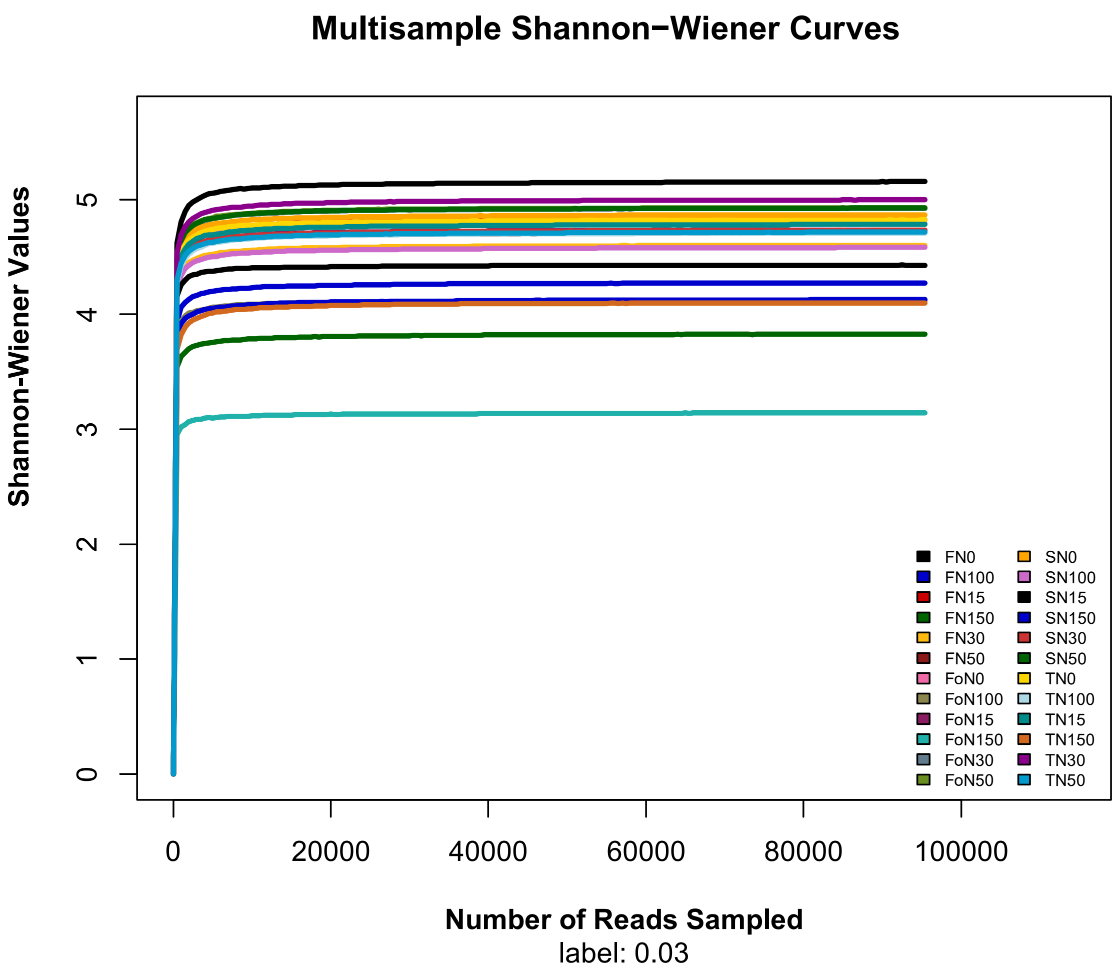

Supplementary Fig. 2 Shannon-Wiener curves. F, Fo, S, and T represent the experimental replicates.

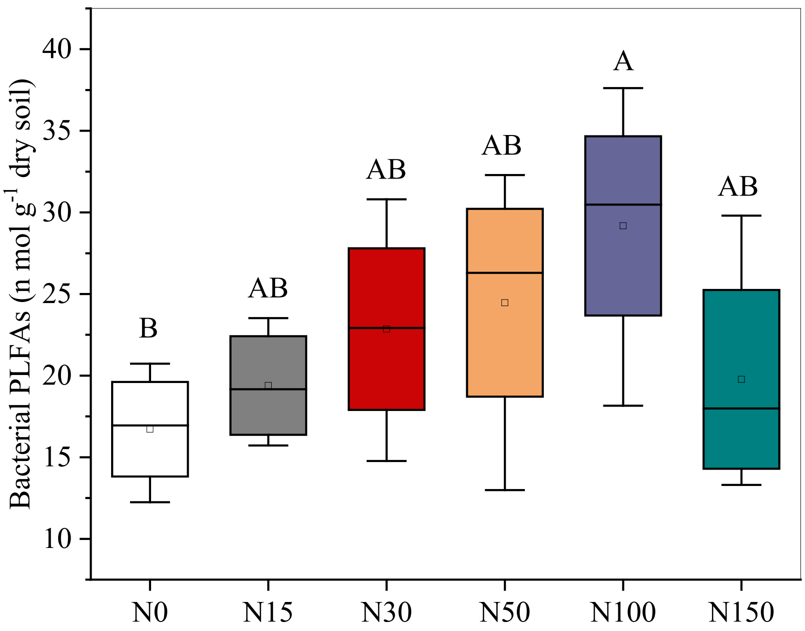

Supplementary Fig. 3 Effects of N addition on the bacterial PLFAs. The different letters above bars indicate significant differences based on the LSD multiple range test (*P*<0.05). The central mark in each box indicates the average value, the central line indicates the median, and the bottom and top edges of the box indicate the 25th and 75th percentiles, respectively. The whiskers extend to the most extreme data points that are not considered outliers, and the outliers are labeled with the '+' symbol.

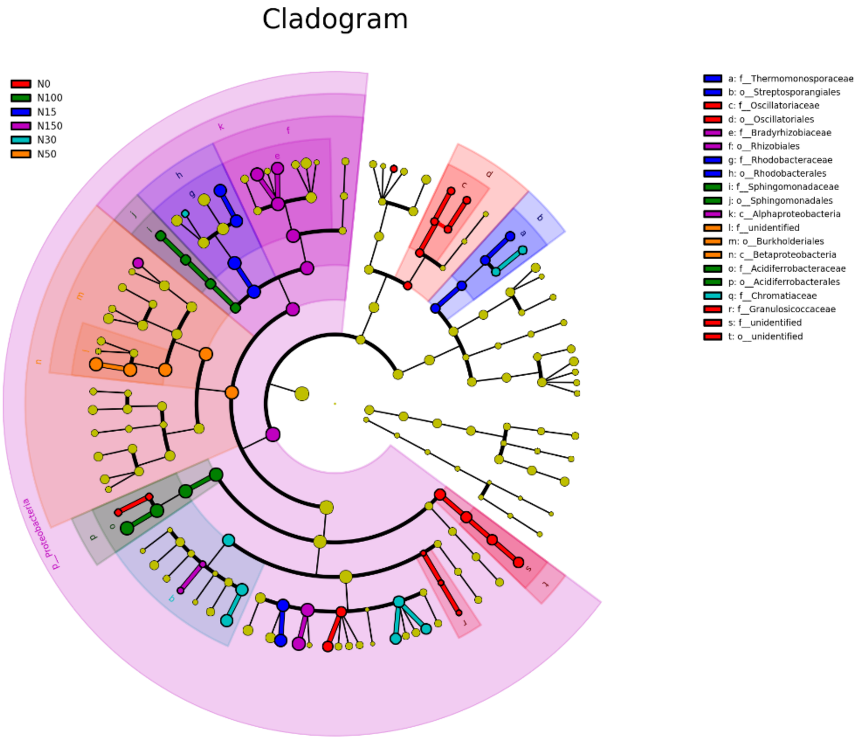

Supplementary Fig. 4 Cladogram showing carbon dioxide-fixing microbial community compositions under different N addition treatments (LEfSe; score=3). The six rings of the cladogram represent the domain (innermost) phylum, class, order, family and genus. The enlarged circles in dark green, blue, and red are differentially abundant taxa identified as taxonomic biomarkers under the different treatments. Lineages with linear discriminant analysis scores with a threshold value of 3.0 were used to identify the degree of differentiation between treatments.

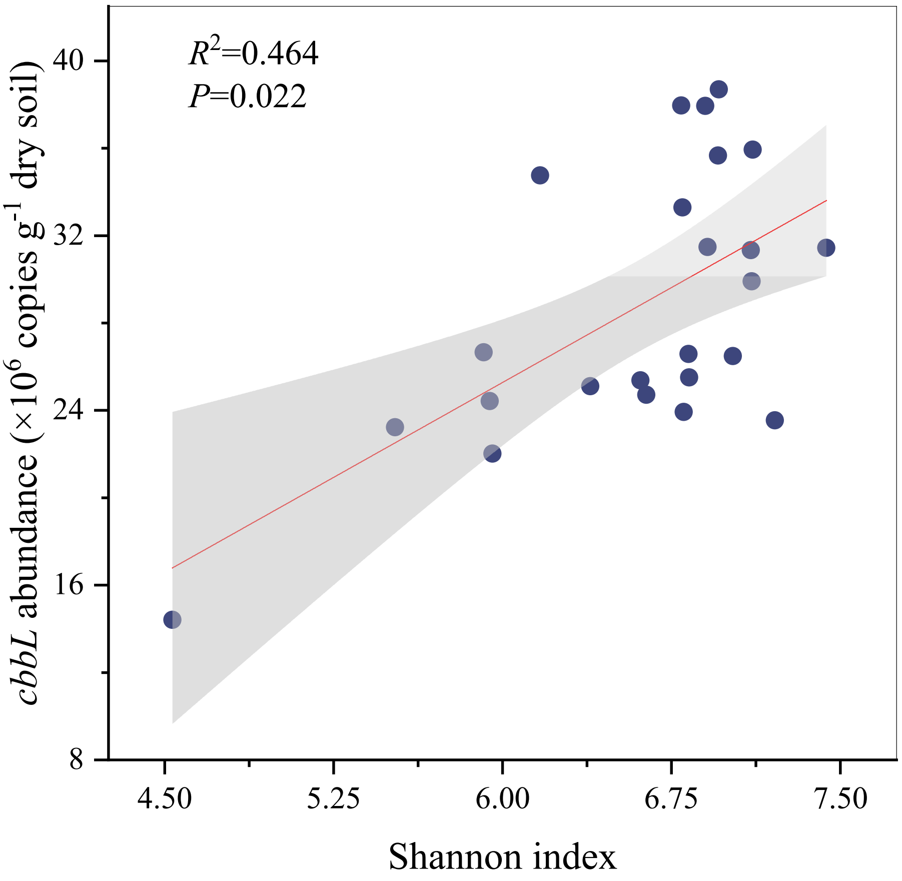

Supplementary Fig. 5 Relationship between the *cbbL* Shannon index values and abundance. The solid red line indicates a significant relationship, and the shaded area shows the 95% confidence interval of the fit.

2.1 Supplementary tables

Supplementary Table 1. Soil physicochemical factors under different N addition levels.

N treatment	Soil organic carbon (g kg^{-1})	Total nitrogen (g kg^{-1})	Total phosphorus (g kg^{-1})	NH_4-N mg kg^{-1}	NO_3-N mg kg^{-1}	pH	N/P	C/N
N0	26.64±0.25a	2.71±0.05b	0.44±0.01a	22.60±1.12d	4.45±0.25c	7.19±0.06a	5.99±0.20c	9.85±0.27ab
N15	27.08±0.28a	2.66±0.02b	0.45±0.01a	21.21±1.25d	3.92±0.14c	6.74±0.02b	5.87±0.13c	10.21±0.09a
N30	27.16±0.49a	2.98±0.14ab	0.45±0.01a	28.80±1.55c	4.28±0.12c	6.56±0.05c	6.76±0.27ab	9.17±0.38bc
N50	27.65±0.70a	2.83±0.19ab	0.43±0.01a	46.70±1.31b	6.16±0.45c	6.64±0.01bc	6.49±0.34bc	9.52±0.36abc
N100	27.72±0.38a	3.09±0.10a	0.45±0.00a	45.63±1.31b	10.81±1.61b	6.33±0.03d	7.07±0.23ab	8.99±0.25bc
N150	26.78±0.52a	3.11±0.11a	0.43±0.01a	71.23±3.78a	15.05±1.07a	5.82±0.07e	7.36±0.23a	8.65±0.30c

Note: Different lowercase letters in the same column indicate significant differences among the treatments at the 0.05 level.

Supplementary Table 2. Results (F values) of an ANOVA of the effects of N addition on bacterial PLFAs, *cbbL* gene abundance, OTUs, the Shannon index and the relative abundance of dominant *cbbL*-containing microbes.

	F	*P*
Bacterial PLFAs	1.796	0.165
Abundance	1.822	0.162
OTUs	2.058	0.125
Shannon index	10.617	0
Chao1	0.745	0.6
Observed species	1.040	0.425
PD whole tree	0.595	0.704
Proteobacteria	10.662	0
Gammaproteobacteria	2.706	0.005
Betaproteobacteria	11.202	0
Alphaproteobacteria	5.000	0.005
Actinobacteria	0.800	0.564
Acidiferrobacterales	6.357	0.001
Chromatiales	0.879	0.515
Burkholderiales	11.049	0
Rhizobiales	11.654	0
Rhodobacterales	14.801	0

Supplementary Table 3. Soil *cbbL*-containing microbes under different N addition levels.

cbbL-containing microbes (relative abundance)	N0	N15	N30	N50	N100	N150
p__Proteobacteria	80.5±1.32bc	79.75±1.25c	78.5±0.87c	81±0.41bc	85±2.55b	91.5±1.56a
c__Gammaproteobacteria	49±1.47ab	46±1.08b	45.5±1.19b	47±1.35b	56.25±2.29ab	54.25±5.81a
c__Betaproteobacteria	19±0.41ab	19.75±0.85a	19.5±0.65a	20.25±0.95a	16±0.41b	11.25±2.02c
c__Alphaproteobacteria	12.75±0.25b	14±0.58b	13.25±1.32b	13.75±1.03b	12.75±0.95b	25.75±5.22a
c__Actinobacteria	0±0a	0.25±0.25a	0±0a	0±0a	0.25±0.25a	0±0a
o__Acidiferrobacterales	27±1.47b	23.75±1.44b	23±0.707b	25±1.78b	34.25±1.80a	26.5±2.10b
o__Chromatiales	21.5±0.29a	22±1.08a	22.5±0.65a	22±1.47a	22±2.04a	27.5±5.20a
o__Burkholderiales	18.75±0.25ab	19.25±0.75a	19.25±0.75a	20±1.08a	15.75±0.479b	11±1.96c
o__Rhizobiales	1±0b	1±0b	0.75±0.25b	1.25±0.25b	2.75±0.75b	20.25±5.50a
o__Rhodobacterales	11.5±0.29ab	13±0.58a	12.75±1.03a	12.75±0.85a	9.25±0.75b	5.5±0.87c

Note: Different lowercase letters in the same row indicate significant differences among the treatments at the 0.05 level.

Supplementary Table 4. Correlation analysis between the bacterial PLFAs and *cbbL* OTUs, abundance and Shannon index values.

		Bacterial PLFAs	*cbbL* OTUs	*cbbL* abundance
cbbL OTUs	R^2	0.065	1	0.620**
	P	0.773		0.001
cbbL abundance	R^2	0.198	0.620**	1
	P	0.364	0.001	
cbbL Shannon index	R^2	0.007	0.825**	0.464*
	P	0.973	0	0.022

Supplementary Table 5. Correlation analyses between soil properties and the *cbbL* OTUs, abundance and Shannon index values.

	cbbL OTUs		*cbbL* abundance		*cbbL* Shannon index	
	R^2	*P* value	R^2	*P* value	R^2	*P* value
SOC	0.145	0.508	0.163	0.457	-0.059	0.788
total N	-0.358	0.086	-0.473*	0.02	-0.541**	0.006
NO_3-N	-0.368	0.077	-0.251	0.236	-0.596**	0.002
NH_4-N	-0.332	0.113	-0.365	0.079	-0.521**	0.009
pH	0.443*	0.03	0.442*	0.031	0.634**	0.001
N/P	-0.325	0.121	-0.460*	0.024	-0.598**	0.002
C/N	0.438*	0.032	0.552**	0.005	0.606**	0.002

SOC indicates soil organic carbon

Supplementary Table 6. Structural equation modeling of the effect of N addition on the *cbbL* diversity (OTUs) and abundance through all plausible interaction pathways. The tables show the unstandardized path coefficients (estimates), standard error of regression weights (S.E.), critical values for the regression weights (C.R.), and levels of significance of the regression weights (*P*). *** indicates $P \leq 0.001$, ** indicates $P \leq 0.01$, and * indicates $P \leq 0.05$.

cbbL OTUs			Estimate	S.E.	C.R.	P
cbbL OTUs	<---	N addition	-0.297	0.105	-2.837	0.005**
NO_3-N	<---	N addition	0.596	0.167	3.563	***
NH_4-N	<---	N addition	0.565	0.172	3.288	0.001***
pH	<---	N addition	-0.539	0.176	-3.067	0.002**
N/P	<---	N addition	0.387	0.192	2.015	0.044*
cbbL OTUs	<---	NO_3-N	-0.374	0.089	-4.203	***
cbbL OTUs	<---	NH_4-N	-0.027	0.095	-0.284	0.777
cbbL OTUs	<---	pH	0.411	0.085	4.841	***
cbbL OTUs	<---	N/P	-0.103	0.085	-1.216	0.224

cbbL abundance			Estimate	S.E.	C.R.	P
cbbL abundance	<---	N addition	-0.388	0.177	-2.188	0.029*
NO_3-N	<---	N addition	0.596	0.167	3.563	***
NH_4-N	<---	N addition	0.565	0.172	3.288	0.001***
pH	<---	N addition	-0.539	0.176	-3.067	0.002**
N/P	<---	N addition	0.387	0.192	2.015	0.044*
cbbL abundance	<---	NO_3-N	0.108	0.151	0.719	0.472
cbbL abundance	<---	NH_4-N	0.204	0.162	1.262	0.207
cbbL abundance	<---	pH	0.432	0.144	3.004	0.003**
cbbL abundance	<---	N/P	-0.478	0.144	-3.326	***
